# Trigonelline Extends the Lifespan of *C. Elegans* and Delays the Progression of Age-Related Diseases by Activating AMPK, DAF-16, and HSF-1

**DOI:** 10.1155/2021/7656834

**Published:** 2021-09-25

**Authors:** Wen-Yu Zeng, Lin Tan, Cong Han, Zhuo-Ya Zheng, Gui-Sheng Wu, Huai-Rong Luo, Su-Lian Li

**Affiliations:** ^1^Affiliated Traditional Chinese Medicine Hospital of Southwest Medical University, Luzhou, Sichuan 646000, China; ^2^Key Laboratory for Aging and Regenerative Medicine, Department of Pharmacology School of Pharmacy, Southwest Medical University, 319 Zhongshan Road, Luzhou, Sichuan 646000, China; ^3^Central Nervous System Drug Key Laboratory of Sichuan Province, Luzhou, Sichuan 646000, China

## Abstract

Trigonelline is the main alkaloid with bioactivity presented in fenugreek, which was used in traditional medicine in Asian countries for centuries. It is reported that trigonelline has anti-inflammatory, anti-oxidant, and anti-pathogenic effects. We are wondering whether trigonelline have anti-aging effect. We found that 50 *μ*M of trigonelline had the best anti-aging activity and could prolong the lifespan of *Caenorhabditis elegans* (*C. elegans*) by about 17.9%. Trigonelline can enhance the oxidative, heat, and pathogenic stress resistance of *C. elegans*. Trigonelline could also delay the development of neurodegenerative diseases, such as AD, PD, and HD, in models of *C. elegans*. Trigonelline could not prolong the lifespan of long-lived worms with loss-of-function mutations in genes regulating energy and nutrition, such as *clk-1*, *isp-1*, *eat-2*, and *rsks-1*. Trigonelline requires *daf-16*, *hsf-1*, and *aak-2* to extend the lifespan of *C. elegans*. Trigonelline can also up-regulate the expression of *daf-16* and *hsf-1* targeted downstream genes, such as *sod-3*, *gst-4*, *hsp-16.1*, and *hsp-12.6*. Our results can be the basis for developing trigonelline-rich products with health benefits, as well as for further research on the pharmacological usage of trigonelline.

## 1. Introduction

The percentage of people aged 65 years and older in developed and developing countries has been steadily increasing. Aging is an intrinsic feature of life and is the greatest risk factor for major age-related disorders, such as diabetes, hypertension, cardiovascular disease, cancer, and neurodegenerative disease [1]. The prevalence of the older population has led to a global burden of age-related disorders. For many years, people have been looking for substances to prevent aging. Experimenting on mammals is time-consuming and expensive. Researchers use cultured human cells to test the anti-aging activity of more substances, but aging is a complex process that is difficult to measure at the cellular level. *Caenorhabditis elegans* (*C. elegans*) is the only multicellular model organism whose somatic developmental lineages have been clearly studied. *C. elegans* has a short life cycle and simple structure. *C. elegans* also has clear genetic background and is easy to manipulate. So, it has become an ideal model for aging research, as well as anti-aging drug screening.

**Figure 1:**
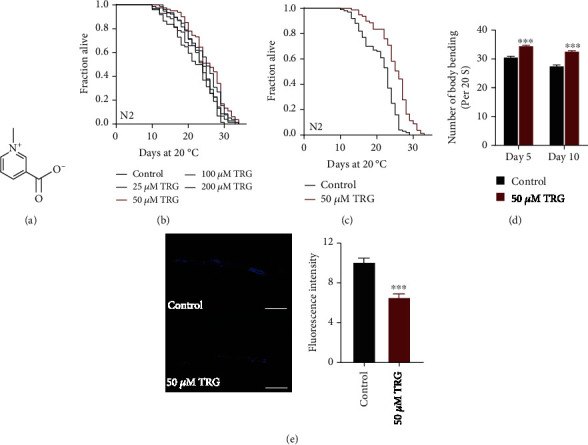
Trigonelline can prolong the lifespan and improve the health of *C. elegans* (a) Chemical structure of trigonelline; (b) Survival curves of wild-type N2 worms raised at 20°C on NGM plates containing either no trigonelline or 25, 50, 100, and 200 *μ*M of trigonelline; (c) Survival curves of wild-type N2 worms treated from hatching and raised at 20°C on NGM plates containing either no trigonelline or 50 *μ*M of trigonelline (*p* <0.001, log-rank test), Statistical details and repeats of these experiments were summarized in Table S1; (d) Body bending of N2 nematode treated with 50 *μ*M of trigonelline for 5 days and 10 days. Figure shows the mean value of three independent experiments, SEM is represented by error line. ∗∗∗ represents *p* <0.001, calculated by two-tailed t-test. Statistical details and repeats of these experiments were summarized in Table S3; (e) Analysis of lipofuscin in Treatment of N2 nematodes by 50 of *μ*M trigonelline on day 10. Relative Fluorescence Intensity was calculated by Image J. Figure shows the mean value of three independent experiments, SEM is represented by error line. ∗∗∗ represents *p* <0.001, calculated by two-tailed t-test. Statistical details and repeats of these experiments were summarized in Table S2.

Fenugreek (*Trigonella foenum-graecum L.*) is an annual forage legume and a traditional spice crop and has been used in traditional medicine in Asian countries for centuries [2]. Modern pharmacological studies have shown that fenugreek has antidiabetic, anticarcinogenic, hypocholesterolemic, antioxidant, and immunological activities [3]. Trigonelline (TRG) is one of the main alkaloids and components with pharmacological activities in dried fenugreek seeds (Figure 1(a)). Trigonelline has been reported to have neuroprotective [4, 5], anti-apoptotic [6, 7], anti-inflammatory [8, 9], anti-oxidant [10], anti-diabetic [11, 12], antihyperglycemic effects [13], anti-degranulation [14], and anti-carcinogenic effect [15, 16]. Among them, the anti-oxidative activity of trigonelline could explain many of its other activities, such as neuroprotective activity [3]. The antioxidant and neuroprotective effects of trigonelline lead us to speculate that trigonelline may play an important role in anti-aging and treating neurodegenerative diseases.

In this study, we investigated whether trigonelline could extend the lifespan and increase the stress tolerances of *C. elegans*. We also tested whether trigonelline could delay the onset of neurodegenerative disease in models of *C. elegans*, as well as determine its possible mechanisms.

## 2. Materials and Methods

### 2.1. Materials

The trigonelline was supplied by Shanghai Yuanye Bio-Technology Ltd (Shanghai,

China, purity ≥95%). Other compounds used in this work were purchased from Sigma-Aldrich (Munich, Germany). Trigonelline was dissolved in ddH_2_O. NGM plates with compound were equilibrated overnight before use.

### 2.2. Worm Strains

All worm strains were provided by the Caenorhabditis Genetic Center (CGC, University of Minnesota, Minneapolis, MN). Except for special cases, worms were cultured in a constant temperature and humidity incubator with a temperature of 20°C and humidity of 60%, on a medium with OP50 *E. coli* food. The nematode strains were as follows: N2 (wild-type), CF1903 *glp-1(e2144) III.*, CF1038 *daf-16 (mu86) I.*, *EU1 skn-1(zu67)IV.*, PS3551 *hsf-1(sy441)I.*, DA1116 *eat-2(ad1116) II.*, MQ887 *isp-1(qm150) IV.*, CB1370 *daf-2(e1370) III.*, RB754 *aak-2(ok524)X.*, RB759 *akt-1(ok525)V.*, VC204 *akt-2(ok393)X.*,VC199 *sir-2.1(ok434)IV.*, CB4876 *clk-1(e2519)III.*, TK22 *mev-1(kn1)III.*, BX165 *nhr-80 (tm1011).*, AA89 *daf-12 (rh274).*, BZ555 *egIs1(dat-1::gfp)*, CF1553 [(pAD76) sod-3::GFP + rol6 (su1006)], CL2166, dvIs19[pAF15(gst-4::GFP::NLS)], SJ4100 (zcIs13[hsp-6::GFP]), SJ4005 zcIs4V (hsp-4::gfp), SJ4058 zcIs9 [hsp-60::GFP + lin-15(+)], CL4176 [(pAF29)myo-3p::A*β*1-42 + (pRF4) rol-6 (su1006)], CL2006 (dvIs2 [pCL12(unc-54/human A*β*1–42 minigene) + pRF4]), NL5901 [unc54p::alphasynuclein::YFP + unc-119(+)], AM140 (rmIs132[unc-54p::Q35::YFP]).

### 2.3. Lifespan Assay

All strains were cultured for 2 - 3 generations without starvation on new NGM plates.

The worms were transferred to NGM plates (containing inactivated OP50 (60°C for 35 min)) with or without trigonelline in late L4 or early adult stage. The plates were supplemented with 20 *μ*M of 5-fluoro-2'-deoxyuridine (FUdR) to inhibit oviposition and hatching. The day when *Caenorhabditis elegans* was transferred to the plates was recorded as the 0th day of the experiment [17]. To ensure that trigonelline retained its potency throughout the entire experiment, worms were transferred to fresh plates with or without trigonelline every other day. In the statistics of lifespan test, if nematodes did not move or did not respond to external mechanical stimulation (such as touching them lightly with insect pickers), they would be recorded as dead. The statistical data of experiments were processed by SPSS 20.0 software, and the results were expressed by Kaplan-Meier survival curve. The *p* value was obtained by log-rank test analysis, while *p* <0.05 indicates statistical significance. Each group of lifespan experiments included at least three independent repeated experiments, and the number of worms in each group was more than 60.

### 2.4. Stress Resistance Assay

For the high temperature resistance test, the synchronized larvae were spread on NGM plates, cultured in an incubator at 20°C, and then transferred to NGM plates with or without trigonelline at the late L4 stage or early adult stage. The worms were transferred to 35°C on the 10th day of adult, and the death of the worm was observed and counted every 2 hours.

For anti-oxidative stress test, pretreatment was the same as high temperature test, the adult worms on the 10th day are transferred to NGM plates containing 20 mM of paraquat, and the death of worms is observed and counted every other day.

For the pathogenic stress test, the pre-treatment was the same as high temperature resistance test. On the 10th day of the adulthood, the worms were transferred to the NGM plates covered with bacteria *Pseudomonas aeruginosa* [17]. The death of worms was observed and recorded every 12 hours.

All statistics and analysis methods of stress assays were the same as the lifespan experiment. The sample size of each experiment was at least 60 worms, and each stress assay contains at least three independent repeated experiments.

### 2.5. Body Bending Behavior Test

The synchronized L1 larva nematodes were spread on NGM plates, cultured in incubator at 20°C. the worms developed to L4 were transferred to experimental plate with or without trigonelline. On the 5th and 10th days of adulthood, the worms were picked up to water droplets and stabilized for 1 minute. Then the bending frequency of the body within 20 seconds were recorded under the microscope, and the back and forth movement of the body was calculated as one bend.

### 2.6. Determination of Reactive Oxygen Species (ROS)

The L1 larvae were spread on NGM plate and cultured in an incubator at 20°C until late L4 or early adulthood. Then, worms were transferred to experimental plates (control group, Trigonelline, NAC, paraquat); After 96 h, the worms were collected and stained with H2DCF-DA probe according to the procedure of ROS detection kit [18]. Then, the nematodes were photographed with fluorescence microscope. The ROS detection experiment includes at least three independent repeated experiments. The sample size of each experiment was at least 30 worms. Image J was used to count the gray value of each nematode. The *p* value was calculated by two-tailed *t*-test.

### 2.7. Determination of Lipofuscin

The preliminary treatment was the same as the lifespan test, the nematodes were collected on the tenth day of the adult and photographed by fluorescence microscope at excitation wavelength of 360-370 nm and emission wavelength of 420-460 nm; The level of lipofuscin in each nematode was quantified by calculating the average pixel intensity of each nematode. Sample size is at least 30 worms.

### 2.8. Fluorescence Quantification

The preliminary treatment of worms for fluorescence quantification was the same as the lifespan test, then the worms of strains Cl2166 (dvis19 [pgst-4p::GFP::nls]) and CF1553 [(pad76) SOD-3::GFP + rol6 (su1006)] were placed at plates containing 20 mM of paraquat for 30 min on the 6th day of adulthood, anesthetized with 2 mM of tetraimidazole hydrochloride. The expression of GFP in these strains was observed by fluorescence microscope. For Transgenic strains SJ4100 (zcIs13[hsp-6::GFP]), SJ4005 zcIs4V (hsp-4::gfp), and SJ4058zcis9 [HSP-60::GFP + Lin-15 (+)], worms were placed at 35°C for 2 h on the 6th day of adults, and was collected with a 1.5 mL centrifuge tube, washed with ddH_2_O for three times to remove OP50, anesthetized with 2 mmol tetraimidazole hydrochloride, and observed the expression of green fluorescent protein with fluorescence microscope.

### 2.9. Age-Related Neurodegenerative Diseases Related Experiments

For worms of strain CL4176(dvis27 (myo-3/abeta1-42/letutr) + prf4 (rol-6 (su1006)), the synchronized larva were spread on the NGM plates with or without trigonelline, incubated at 15°C for 36 hours, then transferred to 23°C for 24 hours, and then counted and photographed every 12 hours.

For worms of strain CL2006 (dvIs2 [pCL12(unc-54/human A*β*1–42 minigene) + pRF4]), the synchronized larvae were spread on NGM plates until they grow into late L4 or early adult stage. Then, the worms were transferred to NGM plate with or without trigonelline, and counted and photographed every day. Nematodes that remain immobile or move only by the head under external mechanical stimulation (such as touching with an insect picker) were defined as paralyzed. The number of worms per group should be above 60.

For the worms of strain AM40 and NL5901, the late L4 larva or early adulthood worms were transferred to experimental plates with or without trigonelline. AM140 nematodes were collected on the 2nd and 4th day of adulthood, and NL5901 nematodes were collected on the 10th day of adulthood. Then, worms were anesthetized with 2 mM of tetraimidazole hydrochloride. The aggregation of *α* -synuclein was captured by Leica fluorescence microscope and analyzed by Image J. The experiment was independently repeated at least three times. The number of worms in each group of experiment is at least 30.

For the worms of transgenic strain BZ555, the L3 larva were transferred to a centrifuge tube containing a mixture of 50 mM of 6-OHDA and 10 mM of ascorbic acid and incubated at 20°C for one hour, and gently shaken every 10 minutes to mix. After one hour, the worms were washed three times with M9 and then placed on a culture plate with and without trigonelline for 72 hours. Finally, we washed the worms with M9, took pictures, and calculated statistics. The experiments were repeated independently at least twice. The number of worms in each group of experiment was at least 30.

### 2.10. Quantitative RT-PCR Assay

About 3,000 synchronized young adult worms were transferred to NGM plates with or without 50 *μ*M of trigonelline and cultured at 20°C for 24 hours. Total RNA was extracted by RNAiso Plus (Takara), converted to cDNA using High Capacity cDNA Reverse Transcription Kit (Applied Biosystems). The quantitative RT-PCR was performed using Power SYBR Green PCR Master Mix (Applied Biosystems) on Quantstudio 6 Flex system. The relative expression levels of genes were calculated by the 2^−*ΔΔ*Ct^ and normalized to the expression of gene cdc-42. The *p* values were calculated using t-test.

## 3. Results

### 3.1. Trigonelline Can Prolong the Lifespan of C. Elegans

To investigate whether trigonelline have anti-aging activity, we treated wild-type N2 worms with 0, 25, 50, 100, and 200 *μ*M of trigonelline, respectively. Our results showed that trigonelline can extend the lifespan of *C. elegans* under various concentrations, among which, 50 *μ*M of trigonelline has the best effect on lifespan extension (Figures 1(b)–1(c)). Lipofuscin is a yellow-brown pigment that accumulates with age [19]. Our results showed that trigonelline could decrease the deposition of lipofuscin (Figure 1(e)). The movement of worms declines as age increases. We found that trigonelline treatment could enhance the movement of worms both at day 5 and day 10 of adulthood (Figure 1(d)).

### 3.2. Trigonelline Can Enhance the Stress Resistance of C. Elegans

**Figure 2:**
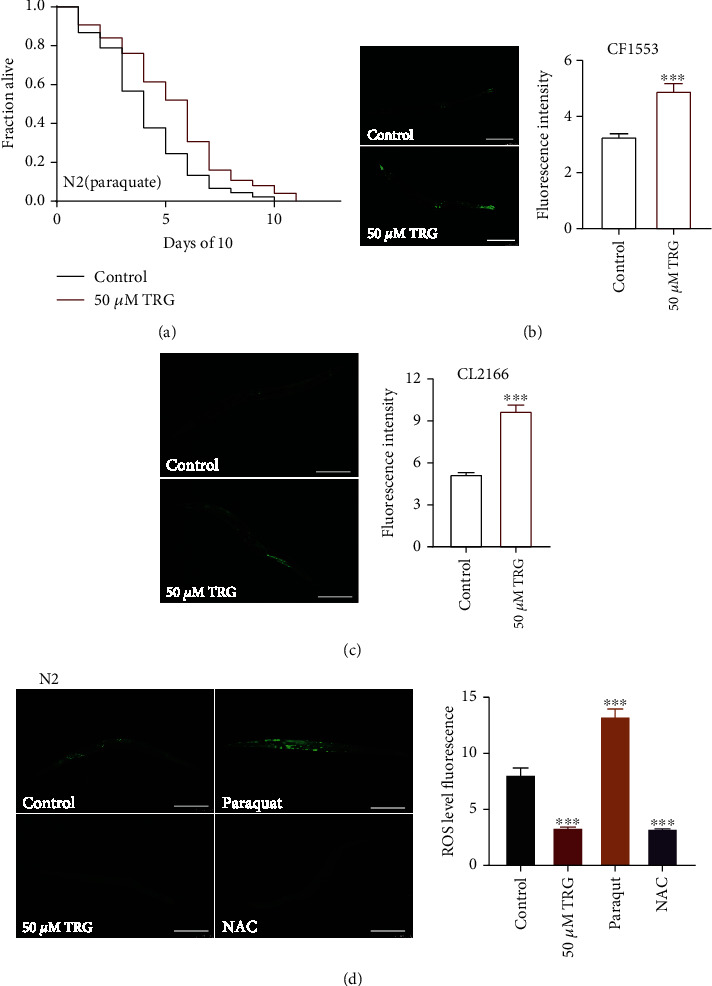
Trigonelline can enhance the oxidative stress resistance of *C. elegans*. The survival percentage of wild-type worms cultured with 20 mM of paraquat under treatment with 50 *μ*M of trigonelline (*p* <0.001, log-rank test). Statistical details and repeats of these experiments were summarized in Table S6; (B-C) The quantification of fluorescence intensity of SOD-3::GFP in CF1553 and GST-4::GFP in CL2166. Trigonelline significantly increased the expression of SOD-3 and GST-4, Fluorescence intensity was calculated by Image J. The bar chart shows the mean value of three independently repeated experiments, and the error line represents SEM. ∗∗∗ represents *p* <0.001, calculated by two-tailed t-test. Statistical details and repeats of these experiments were summarized in Table S5; (D) ROS content detection of wild type N2 treated with 50 *μ*M of trigonelline, while 10 mM of paraquat and 2 mM of NAC were used as negative control and positive control, respectively. Statistical details and repeats of these experiments were summarized in Table S7.

Genetic and dietary interference that extend lifespan usually could also enhance the stress resistance of *C. elegans* [20]. So, we investigated if trigonelline could increase the resistance of *C. elegans* to oxidative, heat, and immune stresses. we detected the changes of ROS content in N2 treated with trigonelline. Trigonelline significantly reduced ROS level in nematodes, and the effect was similar to the antioxidant NAC (Figure 2(d)). Moreover, trigonelline increased the survival rate of N2 worms under oxidative stress induced by 20 mM of paraquat (Figure 2(a)). Trigonelline treatment significantly increased the mRNA and protein expression levels of genes encoding superoxide dismutase 3 (SOD-3) and glutathione S-transferase 4 (GST-4) (Figures 2(b)–2(c)). These results suggest that trigonelline had strong antioxidant effects.

**Figure 3:**
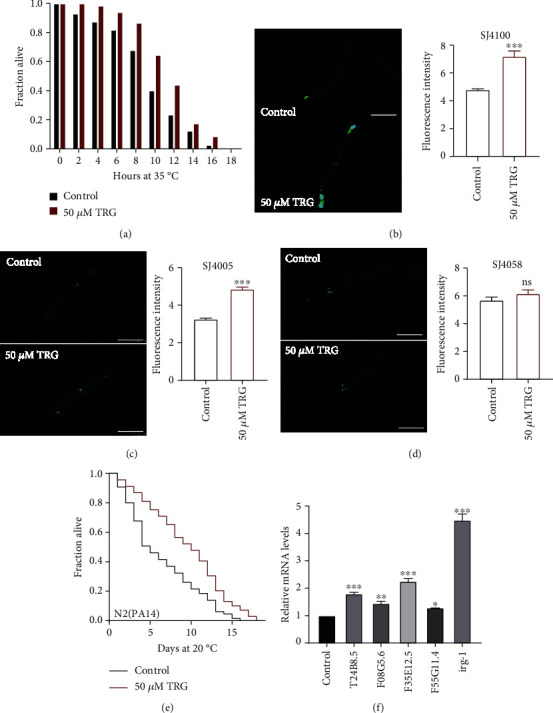
Trigonelline can enhance the ability of nematode to resist heat and pathogenic stresses (a) The survival percentage of wild-type worms treated with or without 50 *μ*M of trigonelline at 35°C (*p* <0.001), Statistic details and repeats of these experiments were summarized in Table S6; (B-D) The quantification of fluorescence intensity of HSP-6::GFP in SJ4100, HSP-4::GFP in SJ4005,HSP-60::GFP, and in SJ4058. Trigonelline significantly increased the expression of HSP-4 and HSP-6, Fluorescence intensity was calculated by Image J. The bar chart shows the mean value of three independently repeated experiments, and the error line represents SEM. ∗∗∗ represents *p* <0.001, calculated by two-tailed t-test. Statistical details and repeats of these experiments were summarized in Table S5; (E) The survival percentage of wild-type worms cultured with *Pseudomonas aeruginosa* under treatment with 50 *μ*M of trigonelline (*p* <0.001, log-rank test). Statistical details and repeats of these experiments were summarized in Table S6; (F) Relative Expression of Immune Genes in L4 Wild-type Worm (N2) Treated with 50 *μ*M of trigonelline for 24 h. Statistical details and repeats of these experiments were summarized in Table S9.

**Figure 4:**
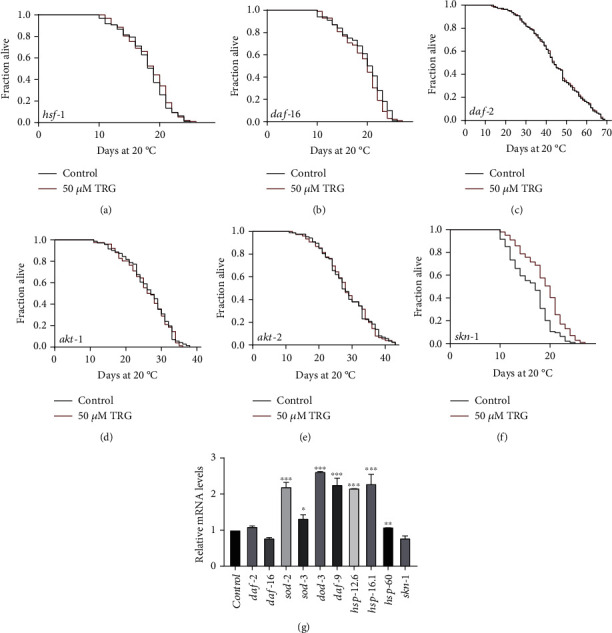
HSF-1 is required for trigonelline to extend nematode lifespan. (A-F) Survival curves of *hsf-1(sy441)I.*, *daf-16(mu86)I.*, *daf-2(e1370)III.*, *akt-1(ok525)V.*, *akt-2(ok393)X.*, and *skn-1(zu67)*, raised at 20°C on NGM plates containing either no trigonelline or 50 *μ*M of trigonelline in lifespan assays (*p* >0.05). Lifespan was analyzed using Kaplan–Meier analysis and *p* values were calculated using log-rank test. Statistic details and repeats of these experiments were summarized in Table S4; (G) Relative expression of downstream *daf-2* genes in L4 wild-type worms (N2) treated with 50 *μ*M of trigonelline for 24 h. Statistical details and repeats of these experiments were summarized in Table S9.

Trigonelline can enhance the survival of *C. elegans* under high temperature of 35°C (Figure 3(a)). Our results showed that trigonelline could increase the mRNA levels of heat shock protein encoding genes, such as *hsp-4*, *hsp-60*, and *hsp-6* (Figure 4(g)). trigonelline treatment could also increase the fluorescence intensity of transgenic nematode strains expressing HSP-4::GFP or HSP-6::GFP (Figures 3(b)–3(d)).

Our results also showed that the survival of N2 nematodes in pathogenic bacteria PA14 was prolonged after being treated with trigonelline for 10 days. Trigonelline treatment increased the mRNA expression levels of immune-related genes, such as *T24B8.5*, *F08G5.6*, *F35E12.5*, *F55G11.4* and *irg-1* (Figures 3(e)–3(f)).

### 3.3. Trigonelline Can Delay the Progression of Age-Related Diseases in C. Elegans Models of AD, PD, and HD

Neurodegenerative diseases are characterized by chronic and progressive decline in neuronal function, which in turn leads to memory deficits, cognitive decline and motor coordination disorder [21]. These diseases include Alzheimer's disease (AD), Parkinson's disease (PD), Huntington's disease (HD), amyotrophy lateral sclerosis (ALS), and spinal muscular atrophy Disease (SMA).

**Figure 5:**
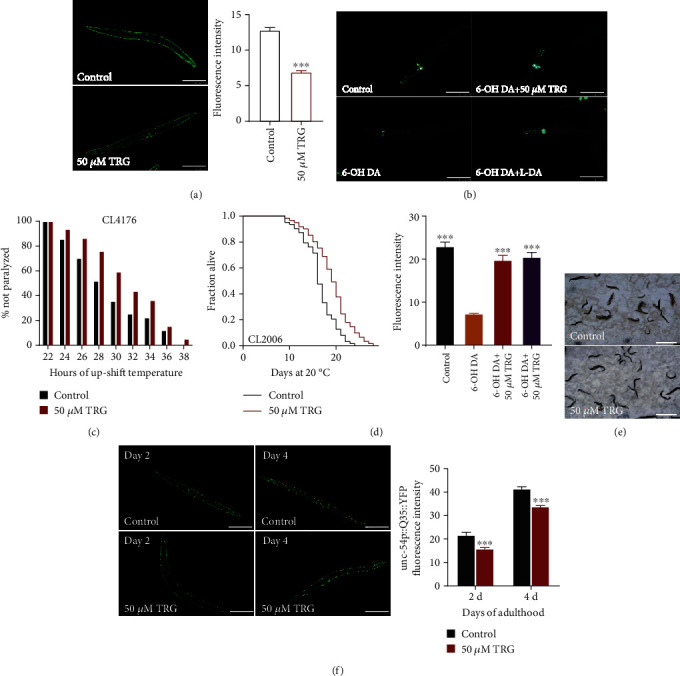
Trigonelline can delay the progression of age-related diseases in *C. elegans* models of AD, PD, and HD Effect of trigonelline on the accumulation of A-synuclein in PD model nematodes; (C-D) Effect of trigonelline on the paralysis rate of AD model in nematode strains CL4176 and CL2006; (E) Effect of trigonelline on the accumulation of Poly-Q in PD model nematodes on day 2 and day 4, Statistical details and repeats of these experiments were summarized in Table S8.

The worm CL4176 expresses the human polypeptide A_*β*1-42_ in the body wall muscle cells at the ambient temperature, resulting in paralysis phenotype of AD-like symptoms in worms. Our results show that trigonelline treatment could delay the onset of paralysis in CL4176 worms (Figure 5(c)). The worm CL2006 also expresses human A_*β*1-42_ driven by the promotor of gene *unc-54* that encoding actin filament. Adult of CL2006 onset paralysis and egg-laying deficiency when raised at 20C. Our results showed that the survival of the nematode CL2006 under progressive paralysis were prolonged after trigonelline treatment (Figures 5(d)–5(e)).

Parkinson's disease (PD) model of nematode NL5901 expresses a human *α*-syn protein fused with yellow fluorescent protein (YFP) in muscle cells of body wall. Trigonelline significantly reduced the *α*-syn aggregation (Figure 5(a)). Worms BZ555 expresses green fluorescent protein (GFP) in the somatic cells and axons of dopamine neurons. Treatment of *Caenorhabditis elegans* with 6-hydroxydopamine (6-OH DA) can selectively induces dopaminergic neuron degeneration. We found that trigonelline can recover neuron injury induced by 6-OH DA, and its activity is like that of the positive drug levodopa (Figure 5(b)).

Huntington's disease (HD) is a fatal autosomal dominant neurodegenerative disease. Its neuropathological features are progressive lesions in basal ganglia, and its clinical manifestations are abnormal movement, cognition, and behavior. AM140 is a transgenic strain expressing yellow fluorescent protein (YFP)-labeled polyglutamine (polyQ). We measured the effect of trigonelline on fluorescence intensity and punctate aggregation in AM140. Trigonelline significantly reduced the accumulation of age-related polyQ in body wall muscles on day 2 and day 4 of adults (Figure 5(f)). Above results shows that trigonelline can improve the performance of worms in neurodegenerative diseases models.

### 3.4. Trigonelline Requires the Transcription Factor DAF-16/FOXO to Extend the Lifespan of C. Elegans

The transcription factor DAF-16/FOXO plays a central role in the regulation of the stress resistance and longevity [22]. So, we examined if trigonelline could extend the lifespan of worms with loss-of-function mutation in *daf-16.* We found that trigonelline could not extend the lifespan of the loss-of-function mutant *daf-16 (mu86) I* (Figure 4(b)). The kinases AKT-1 and AKT-2 act at upstream of DAF-16 in the insulin signaling pathway. We found that trigonelline also could not extend the lifespan of worms with loss-of-function mutations in *akt-1* and *akt-2*. (Figures 4(c)–4(e)).

The transcription factor SKN-1 is the homologue of mammalian NF-E2-related factor 2 (Nrf2), plays a central role in the induction of cytoprotective genes in response to oxidative stress [23]. We found that trigonelline could extend the lifespan of worms with a loss-of-function mutation in *skn-1* (Figure 4(f)).

The conserved heat stress transcription factor HSF-1 regulates the expression of cellular chaperone genes to maintain the proteostasis from external environmental stresses and internal age-related damages [24]. We investigated if trigonelline need HSF to extend the lifespan of worms. Our results showed that trigonelline could not extend the lifespan of worms with loss-of-function mutant in *hsf-1* (Figure 4(a)).

### 3.5. Trigonelline Could Not Further Extend the Lifespan of Worms with Mutations in the Genes Regulating Energy Production and Nutrition Uptake

**Figure 6:**
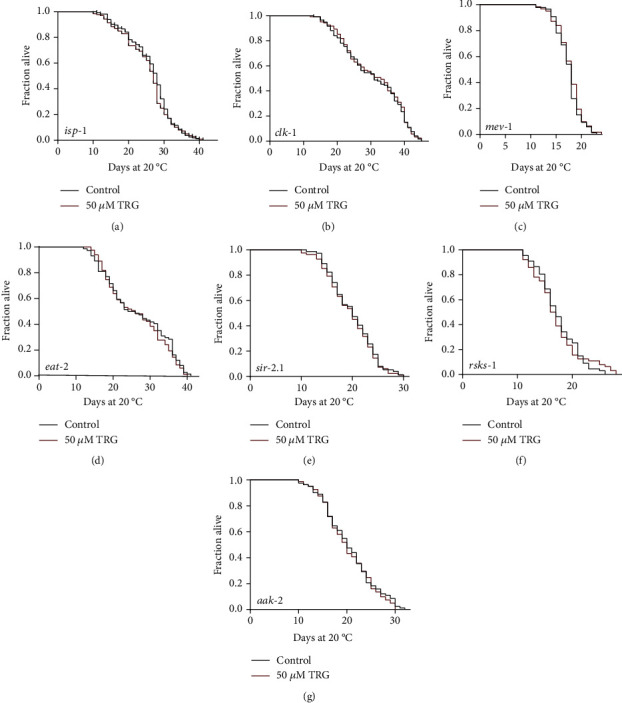
Trigonelline prolongs lifespan through mitochondrial signaling pathways (A-G) Survival curves of *isp-1 (qm150)*, *clk-1(e2519)III.*, and *mev-1(kn1)III.* raised at 20°C on NGM plates containing either no trigonelline or 50 *μ*M trigonelline in lifespan assays (*p* >0.05). Lifespan was analyzed using Kaplan-Meier analysis and *p* values were calculated using log-rank test. Statistic details and repeats of these experiments were summarized in Table S4.

The decline in mitochondrial energy production and the damage caused by mitochondrial reactive oxygen species (ROS) are the main causes of aging [25]. The genes involved in mitochondrial electron transport are also the regulators of oxidative stress responses [26]. The gene *clk-1* encodes a mitochondrial enzyme or regulatory molecule in the ubiquinone biosynthetic pathway. The gene *mev-1* encodes a homolog of the C subunit of the human succinate dehydrogenase complex, while *isp-1* encodes the ubiquitin-cytochrome c reductase, the homolog of Rieske iron-sulfur polypeptide 1. We investigated if interruption of these genes would affect the effect of trigonelline on lifespan extension. Our results showed that trigonelline could not extend the lifespan of worms carrying the loss-of-function mutations of the three genes (Figures 6(a)–6(c)).

Dietary restriction can affect a variety of metabolic pathways and stress resistance pathways, including reducing cell damage induced by oxidative stress, improving mitochondrial function, thereby reducing age-related mitochondria function decline [27]. DA1116 *eat-2(ad1116) II.* has slowed pumping rate, mimics dietary restriction phenotypes and presents extended lifespan compared with wild type N2. We found that trigonelline could not extend the lifespan of DA1116 *eat-2(ad1116) II..* Dietary restriction regulates the activity of NAD-dependent protein deacetylase SIR-2.1, 5'-AMP-activated protein kinase (AMPK) catalytic subunit 2, and Ribosomal protein S6 kinase beta (encoded by gene *rsks-1*). We found that trigonelline treatment could not extend the lifespan of DA1116 *eat-2(ad1116) II* and worms with loss-of-function mutation in *sir-2*, which encodes NAD-dependent protein deacetylase (Figures 6(d)–6(e)). Gene aak-2 encodes 5'-AMP-activated protein kinase catalytic subunit 2, which plays a role in the regulation of lifespan, dauer larval development and protein secretion. Ribosomal protein S6 kinase beta is a key factor of the target-of-rapamycin (TOR) pathway, regulates nematode lifespan and iron homeostasis. Our experiments show that trigonelline could not extend the lifespan of worms with loss-of-function mutations in genes *aak-2* and *rsks-1* (Figures 6(f)–6(g)).

## 4. Discussion

Trigonelline, a plant alkaloid, is the main active ingredient of coffee and fenugreek [2, 28]. We found that trigonelline at different concentrations can prolong the lifespan of *C. elegans*. Trigonelline treatment could enhance the locomotor ability of *C. elegans*, reduce the accumulation of lipofuscin in the body. Trigonelline can increase the heat, oxidative, and pathogenic stress resistance of worms. Trigonelline could also delay the progression of age-related diseases in *C. elegans* models of AD, PD, and HD.

The transcription factor DAF-16/FOXO plays a central role in the regulation of the stress resistance and longevity [22]. The kinases AKT-1 and AKT-2 act at upstream of DAF-16 in the insulin signaling pathway [29]. The anti-aging activity of trigonelline requires the transcription factor DAF-16/FOXO. Trigonelline also could not further extend the lifespan of worms with the loss-of-function mutations in *akt-1* and *akt-2*. Since the mutants of *akt-1* and *akt-2* are long-lived, it was uncertain if trigonelline act on the upstream of AKT or the effect of Trigonelline on lifespan extension was not strong enough to show significant difference from the mutants of *akt-1* and *akt-2*. The transcription factor SKN-1plays a central role in the induction of cytoprotective genes in response to oxidative stress [23]. The conserved heat stress transcription factor HSF-1 regulates the expression of cellular chaperone genes to maintain the proteostasis [24]. Both SKN-1 and HSF-1 are the downstream target of DAF-16. Our results showed that trigonelline require HSF-1, but not SKN-1 to extend the lifespan of *C. elegans*, indicating trigonelline might act through stabilizing the proteostasis of *C. elegans*.

We found that trigonelline could not extend the lifespan of the worms with loss-of-function mutations in the genes *clk-1*, *mev-1*, *isp-1*, *eat-2*, *sir-2.1*, *aak-2*, and *rsks-1*. The genes *clk-1*, *mev-1*, and *isp-1* encode mitochondrial proteins regulating energy production, while *eat-2* regulates food uptake. AMPK senses the energy level and passes the information to TOR, which regulates the activity of ribosomal protein S6 kinase beta [27]. TOR is the central energy and nutrition sensor and plays a critical role in growth, metabolism, autophagy, and proteostasis [30]. Above results showed that trigonelline requires *aak-2* and extends the lifespan of *C. elegans* through the energy pathway.

A common etiology of neurodegenerative diseases, such as AD, PD, and HD, is the abnormal deposition of proteins [31]. Our results showed that trigonelline could delay the onset of neurodegenerative diseases in various models of *C. elegans*. Trigonelline could delay the paralysis rate of Alzheimer's disease, reduce the accumulation of *α*-syn in the worm muscle of Parkinson's model, repair the 6-OH DA-induced neuronal damage, and reduce the accumulation of Poly Q in AM140 worm of Huntington model. These results showed that trigonelline could improve the proteostasis of *C. elegans*. This is consistent with that trigonelline requires HSF-1 and AMPK for anti-aging effects, because both HSF-1 and AMPK could regulate the protein homeostasis of *C. elegans*.

In summary, trigonelline treatment can prolong the healthy lifespan, increase the stress tolerance of *C. elegans*, and delay the development of neurodegenerative diseases in models of nematodes. Finally, trigonelline appear to require transcription factor DAF-16/FOXO, HSF-1 and AMPK to maintain the protein homeostasis and extend the lifespan of *C. elegans*. Our results can be the basis for developing trigonelline-rich products with health benefits, as well as for further research on the pharmacological usage of trigonelline.

## Data Availability

All the figures and tables used to support the findings of this study are included within the article and supplementary materials.

## Supplementary Materials

Supplementary Materials Table S1. Lifespan of wild-type nematode (N2) treated with TRG in different concentrations. Table S2. Effect of TRG on lipofuscin in wild-type nematode (N2). Table S3. Effect of TRG on body movement in wild-type nematode (N2). Table S4. Effect of TRG on lifespan of mutant nematode. Table S5. Effect of TRG on DAF-16 nuclear localization and expression of HSP-4, HSP-6, HSP-60, GST-4 and SOD-3 in mutant nematode. Table S6. Effect of TRG on resistance to bacteria, high temperature and oxidation in wild-type nematode (N2). Table S7. Effects of TRG on age-related diseases. Table S8. Effect of TRG on gene expression at mRNA level in nematode. Table S9. Primers used for the analysis of mRNA expression levels in nematode.
