# Supplementary material for: Trigonelline Extends the Lifespan of C. Elegans and Delays the Progression of Age-Related Diseases by Activating AMPK, DAF-16, and HSF-1
Source: Oxid Med Cell Longev. 2021 Sep 25;2021:7656834. doi: 10.1155/2021/7656834 (PMC8487828; doi:10.1155/2021/7656834)
Supplement: Supplementary Materials — Table S1. Lifespan of wild-type nematode (N2) treated with TRG in different concentrations. Table S2. Effect of TRG on lipofuscin in wild-type nematode (N2). Table S3. Effect of TRG on body movement in wild-type nematode (N2). Table S4. Effect of TRG on lifespan of mutant nematode. Table S5. Effect of TRG on DAF-16 nuclear localization and expression of HSP-4, HSP-6, HSP-60, GST-4 and SOD-3 in mutant nematode. Table S6. Effect of TRG on resistance to bacteria, high temperature and oxidation in wild-type nematode (N2). Table S7. Effects of TRG on age-related diseases. Table S8. Effect of TRG on gene expression at mRNA level in nematode. Table S9. Primers used for the analysis of mRNA expression levels in nematode. [file 7656834.f1.docx]

**Trigonelline Extends the Lifespan of *C. elegans* and Delays the Progression of Age-Related Diseases by Activating AMPK, DAF-16, and HSF-1**

Wen-Yu Zeng^1^, Lin Tan^2^, Cong Han^1^, Zhuo-Ya Zheng^2^, Gui-Sheng Wu^2, 3^, Huai-Rong Luo^2,3,*^, Su-Lian Li^1,*^

1 Affiliated Traditional Chinese Medicine Hospital of Southwest Medical University, Luzhou, Sichuan, 646000, China.
2 Key Laboratory for Aging and Regenerative Medicine, Department of Pharmacology School of Pharmacy, Southwest Medical University, 319 Zhongshan Road, Luzhou, Sichuan, 646000, China.

3 Central Nervous System Drug Key Laboratory of Sichuan Province, Luzhou, Sichuan, 646000, China.

* Corresponding author: Dr. Huai-Rong Luo, Su-Lian Li;

Key Laboratory for Aging and Regenerative Medicine, Department of Pharmacology

School of Pharmacy, Southwest Medical University

319 Zhongshan Road, Luzhou, Sichuan 646000, China

Phone: +86 830-3160842; Fax: +86 830-3160842

E-mail address: [lhr@swmu.edu.cn](mailto:lhr@swmu.edu.cn), [lyzyhb@163.com](mailto:lyzyhb@163.com)

ORCID: <https://orcid.org/0000-0001-8912-6694>

**Supplementary materials**

**Contents:**

**Table S1. Lifespan of wild-type nematode (N2) treated with TRG in different concentrations.**

**Table S2. Effect of TRG on lipofuscin in wild-type nematode (N2).**

**Table S3. Effect of TRG on body movement in wild-type nematode (N2).**

**Table S4. Effect of TRG on lifespan of mutant nematode.**

**Table S5. Effect of TRG on DAF-16** [**nuclear**](javascript:;) [**localization**](javascript:;)**and expression of HSP-4, HSP-6, HSP-60, GST-4 and SOD-3 in mutant nematode*.***

**Table S6. Effect of TRG on resistance to bacteria, high temperature and oxidation in wild-type nematode (N2).**

**Table S7. Effects of TRG on age-related diseases.**

**Table S8. Effect of TRG on gene expression at mRNA level in nematode.**

**Table S9. Primers used for the analysis of mRNA expression levels in nematode.**

**Table S1. Lifespan of wild-type nematode (N2) treated with TRG in different concentrations.**

| Figure  1b Concentration |  | Control  (0 μM) | 25 μM  TRG | 50 μM  TRG | 100 μM  TRG | 200 μM  TRG |
| --- | --- | --- | --- | --- | --- | --- |
| strain |  | N2 | N2 | N2 | N2 | N2 |
| Treatments |  | 20℃/OP50  (dead) | 20℃/OP50  (dead) | 20℃/OP50  (dead) | 20℃/OP50  (dead) | 20℃/OP50  (dead) |
| Mean±SEM | EXP.1  EXP.2  EXP.3 | 21.070±0.463  20.392±0.562  21.689±0.669 | 23.048±0.508  22.616±0.528  24.179±0.625 | 24.848±0.501  23.469±0.511  25.220±0.570 | 22.671±0.510  22.395±0.598  23.250±0.553 | 21.104±0.421  21.837±0.524  22.714±0.680 |
| *P* value  VS control | EXP.1  EXP.2  EXP.3 |  | <0.001  <0.001  <0.001 | <0.0001  <0.0001  <0.0001 | <0.001  <0.001  <0.001 | #  <0.05  <0.05 |
| N | EXP.1  EXP.2  EXP.3 | 100  97  74 | 83  99  78 | 79  113  82 | 76  86  76 | 106  98  70 |
| change in mean lifespan | EXP.1  EXP.2  EXP.3 |  | 9.388%  10.906%  11.480% | 17.931%  15.089%  16.280% | 7.598%  9.822%  7.197% | 0.161%  7.086%  4.726% |

*p*-value was analyzed by logRankTest.

N: number of dead worms.

*P* <0.05 indicated that the experiment was statistically significant, while *P* >0.05 indicated that the experiment was not statistically significant.

**Table S2. Effect of TRG on lipofuscin in wild-type nematode (N2).**

| Figure  1E | Strain | Treatments | Pigment Mean±SEM | P value  VS  control | N |
| --- | --- | --- | --- | --- | --- |
|  | N2（WT） | OP50(dead) |  |  |  |
|  | EXP.1  EXP.1 | 20℃/control  20℃/50μM  TRG | 9.391±0.451  6.529±0.417 | <0.0001 | 30  30 |
|  | EXP.2  EXP.2 | 20℃/control  20℃/50μM  TRG | 10.091±0.522  6.555±0.463 | <0.0001 | 30  30 |
|  | EXP.3  EXP.3 | 20℃/control  20℃/50μM  TRG | 9.491±0.571  5.519±0.328 | <0.0001 | 30  32 |

On the 10th day of the adult worm, the accumulation of lipofuscin in intestinal tissues of the treated and untreated nematodes was photographed and counted. Fluorescence intensity was analyzed by Image J, and *p* value was calculated by two-tailed t-test, where *P* <0.05 indicated that the experiment was statistically significant.

**Table S3. Effect of TRG on body movement in wild-type nematode (N2).**

| Figure  1D | Strain | Treatment | Number of body bending Mean ± SEM | P value  VS  control | N | | Number of body bending  Mean ± SEM | P value  VS  control | | N | |
| --- | --- | --- | --- | --- | --- | --- | --- | --- | --- | --- | --- |
|  | N2 | OP50(dead) | 5 day of adult | | | | 10 day of adult | | | | |
|  | EXP.1  EXP.1 | 20℃/control  20℃/50μM  TRG | 33.286±0.682  34.243±0.841 | <0.05 | | 28  28 | 25.179± 0.692  28.897± 0.874 | | <0.001 | | 28  28 |
|  | EXP.2  EXP.2 | 20℃/control  20℃/50μM  TRG | 32.036±0.581  34.241±0.450 | <0.001 | | 28  28 | 26.036±0.964  30.310±0.827 | | <0.001 | | 28  28 |
|  | EXP.3  EXP.3 | 20℃/control  20℃/50μM TRG | 30.775±0.580  34.675±0.474 | <0.001 | | 40  40 | 27.700±0.612  32.800±0.487 | | <0.001 | | 40  40 |

The body movement count time was 20 seconds per nematode. N was the experimental sample size, and P value was calculated by two-tailed t-test. *P* <0.05 indicated that the experiment was statistically significant

**Table S4. Effect of TRG on lifespan of mutant nematode.**

| Figure | Strain | Treatment | Mean lifespan | P value | change in mean lifespan | N |
| --- | --- | --- | --- | --- | --- | --- |
|  |  |  | ±SEM | VS |  |  |
|  |  |  | (hours) | control |  |  |
| 5C | **CF1370** |  | Day |  |  |  |
|  | ***daf-2(e1370)Ⅲ.*** | OP50(dead) |  |  |  |  |
|  | EXP.1 | 20℃/control | 44.282±1.286 | 0.868 | # | 110 |
|  | EXP.1 | 20℃/50μM TRG | 44.477±1.303 |  |  | 109 |
|  | EXP.2 | 20℃/control | 45.279±1.182 | 0.854 | # | 111 |
|  | EXP.2 | 20℃/50μM TRG | 44.566±1.198 |  |  | 113 |
|  | EXP.3 | 20℃/control | 46.131±1.280 | 0.786 | # | 99 |
|  | EXP.3 | 20℃/50μM TRG | 47.310±1.251 |  |  | 100 |
|  |  |  |  |  |  |  |
| 5D | **RB759** |  |  |  |  |  |
|  | ***akt-1(ok525)V.*** | OP50(dead) |  |  |  |  |
|  | EXP.1 | 20℃/control | 28.054±0.735 | 0.68 | # | 92 |
|  | EXP.1 | 20℃/50μM | 28.134±0.702 |  |  | 97 |
|  |  | TRG |  |  |  |  |
|  | EXP.2 | 20℃/control | 27.782±0.782 | 0.979 | # | 78 |
|  | EXP.2 | 20℃/50μM | 27.200±0.886 |  |  | 65 |
|  |  | TRG |  |  |  |  |
|  | EXP.3 | 20℃/control | 27.706±0.891 | 0.408 | # | 68 |
|  | EXP.3 | 20℃/50μM | 27.867±1.132 |  |  | 60 |
|  |  | TRG |  |  |  |  |
|  | EXP.4 | 20℃/control | 28.379±0.791 | 0.917 | # | 87 |
|  | EXP.4 | 20℃/50μM | 28.395±0.841 |  |  | 76 |
|  |  | TRG |  |  |  |  |
| 5E | **VC204 *akt-2(ok393)X*** |  |  |  |  |  |
|  | EXP.1 | OP50(dead) |  |  |  |  |
|  | EXP.1 | 20℃/control | 25.848±0.751 | 0.456 | # | 66 |
|  |  | 20℃/50μM | 26.520±0.711 |  |  | 75 |
|  |  | TRG |  |  |  |  |
|  | EXP.2 | 20℃/control | 26.368±0.851 | 0.587 | # | 76 |
|  | EXP.2 | 20℃/50μM | 26.831±0.923 |  |  | 71 |
|  |  | TRG |  |  |  |  |
|  | EXP.3 | 20℃/control | 26.352±0.750 | 0.776 | # | 71 |
|  | EXP.3 | 20℃/50μM | 26.039±0.721 |  |  | 76 |
|  |  | TRG |  |  |  |  |
| 5B | **CF1038** |  |  |  |  |  |
|  | ***Daf-16(mu86)Ⅰ.*** | OP50(dead) |  |  |  |  |
|  | EXP.1 | 20℃/control | 18.854±0.433 | 0.361 | # | 89 |
|  | EXP.1 | 20℃/50μM | 18.145±0.535 |  |  | 69 |
|  |  | TRG |  |  |  |  |
|  | EXP.2 | 20℃/control | 19.530±0.433 | 0.122 | # | 100 |
|  | EXP.2 | 20℃/50μM | 18.960±0.405 |  |  | 99 |
|  |  | TRG |  |  |  |  |
|  | EXP.3 | 20℃/control | 18.978±0.477 | 0.598 | # | 90 |
|  | EXP.3 | 20℃/50μM | 19.093±0.454 |  |  | 97 |
|  |  | TRG |  |  |  |  |
| 5A | **PS3551** |  |  |  |  |  |
|  | ***hsf-1(sy441)Ⅰ.*** | OP50(dead) |  |  |  |  |
|  | EXP.1 | 20℃/control | 18.041±0.365 | 0.432 | # | 97 |
|  | EXP.1 | 20℃/50μM | 18.340±0.366 |  |  | 97 |
|  |  | TRG |  |  |  |  |
|  | EXP.2 | 20℃/control | 17.935±0.459 | 0.581 | # | 77 |
|  | EXP.2 | 20℃/50μM | 18.213±0.460 |  |  | 80 |
|  |  | TRG |  |  |  |  |
|  | EXP.3 | 20℃/control | 18.677±0.402 | 0.455 | # | 96 |
|  | EXP.3 | 20℃/50μM | 17.361±0.510 |  |  | 83 |
|  |  | TRG |  |  |  |  |
|  | EXP.4 | 20℃/control | 17.654±0.444 | 0.604 | # | 81 |
|  | EXP.4 | 20℃/50μM | 17.260±0.455 |  |  | 77 |
|  |  | TRG |  |  |  |  |
| 5F | **EU1** |  |  |  |  |  |
|  | ***skn-1(zu67)*** | OP50(dead) |  |  |  |  |
|  | EXP.1 | 20℃/control | 16.117±0.411 | 0 | 16.635 | 94 |
|  | EXP.1 | 20℃/50μM | 18.798±0.415 |  |  | 99 |
|  |  | TRG |  |  |  |  |
|  | EXP.2 | 20℃/control | 17.578±0.679 | 0.009 | 13.557 | 64 |
|  | EXP.2 | 20℃/50μM | 19.961±0.552 |  |  | 77 |
|  |  | TRG |  |  |  |  |
|  | EXP.3 | 20℃/control | 17.069±0.653 | 0.073 | 15.168 | 72 |
|  | EXP.3 | 20℃/50μM | 19.658±0.584 |  |  | 79 |
|  |  | TRG |  |  |  |  |
| 6A | **MQ887** |  |  |  |  |  |
|  | ***isp-1(qm150)IV*** | OP50(dead) |  |  |  |  |
|  | EXP.1 | 20℃/control | 24.882±0.719 | 0.744 | # | 119 |
|  | EXP.1 | 20℃/50μM | 25.064±0.647 |  |  | 125 |
|  |  | TRG |  |  |  |  |
|  | EXP.2 | 20℃/control | 26.364±0.495 | 0.452 | # | 165 |
|  | EXP.2 | 20℃/50μM | 25.593±0.571 |  |  | 140 |
|  |  | TRG |  |  |  |  |
|  | EXP.3 | 20℃/control | 24.007±0.606 | 0.08 | # | 140 |
|  | EXP.3 | 20℃/50μM | 25.405±0.597 |  |  | 153 |
|  |  | TRG |  |  |  |  |
| 6B | **CB4876** |  |  |  |  |  |
|  | ***clk-1(e2519)III.*** | OP50(dead) |  |  |  |  |
|  | EXP.1 | 20℃/control | 31.770±1.130 | 0.383 | # | 61 |
|  | EXP.1 | 20℃/50μM | 32.311±1.156 |  |  | 61 |
|  |  | TRG |  |  |  |  |
|  | EXP.2 | 20℃/control | 30.800±1.017 | 0.762 | # | 65 |
|  | EXP.2 | 20℃/50μM | 30.803±1.047 |  |  | 66 |
|  |  | TRG |  |  |  |  |
|  | EXP.3 | 20℃/control | 30.647±0.840 | 0.83 | # | 119 |
|  | EXP.3 | 20℃/50μM | 30.984±0.820 |  |  | 122 |
|  |  | TRG |  |  |  |  |
| 6C | **TK22** |  |  |  |  |  |
|  | ***mev-1(kn1)III.*** | OP50(dead) |  |  |  |  |
|  | EXP.1 | 20℃/control | 17.500±0.304 | 0.264 | # | 80 |
|  | EXP.1 | 20℃/50μM | 17.141±0.295 |  |  | 78 |
|  |  | TRG |  |  |  |  |
|  | EXP.2 | 20℃/control | 17.425±0.249 | 0.406 | # | 87 |
|  | EXP.2 | 20℃/50μM | 17.661±0.317 |  |  | 62 |
|  |  | TRG |  |  |  |  |
|  | EXP.3 | 20℃/control | 17.101±0.357 | 0.483 | # | 79 |
|  | EXP.3 | 20℃/50μM | 16.608±0.370 |  |  | 74 |
|  |  | TRG |  |  |  |  |
| 6D | **DA1116 *eat-2(ad1116)II.*** |  |  |  |  |  |
|  | EXP.1 | OP50(dead) |  |  |  |  |
|  | EXP.1 | 20℃/control | 25.046±1.008 | 0.393 | # | 65 |
|  |  | 20℃/50μM | 26.148±0.928 |  |  | 76 |
|  |  | TRG |  |  |  |  |
|  | EXP.2 | 20℃/control | 24.192±0.836 | 0.4 | # | 78 |
|  | EXP.2 | 20℃/50μM | 25.015±0.963 |  |  | 67 |
|  |  | TRG |  |  |  |  |
|  | EXP.3 | 20℃/control | 26.541±1.032 | 0.458 | # | 74 |
|  | EXP.3 | 20℃/50μM | 26.253±0.900 |  |  | 83 |
|  |  | TRG |  |  |  |  |
| 6E | **VC199 *sir-2.1(ok434)I.*** |  |  |  |  |  |
|  | EXP.1 | OP50(dead) |  |  |  |  |
|  | EXP.1 | 20℃/control | 19.956±0.519 | 0.618 | # | 68 |
|  |  | 20℃/50μM | 19.710±0.519 |  |  | 69 |
|  |  | TRG |  |  |  |  |
|  | EXP.2 | 20℃/control | 18.984±0.478 | 0.327 | # | 64 |
|  | EXP.2 | 20℃/50μM | 19.507±0.465 |  |  | 71 |
|  |  | TRG |  |  |  |  |
|  | EXP.3 | 20℃/control | 20.068±0.510 | 0.618 | # | 74 |
|  | EXP.3 | 20℃/50μM | 19.634±0.501 |  |  | 82 |
|  |  | TRG |  |  |  |  |
| 6G | **RB754** |  |  |  |  |  |
|  | ***aak-2(ok524)X.*** | OP50(dead) |  |  |  |  |
|  | EXP.1 | 20℃/control | 17.821±0.464 | 0.809 | # | 78 |
|  | EXP.1 | 20℃/50μM | 17.500±0.506 |  |  | 72 |
|  |  | TRG |  |  |  |  |
|  | EXP.2 | 20℃/control | 17.367±0.397 | 0.563 | # | 109 |
|  | EXP.2 | 20℃/50μM | 17.692±0.467 |  |  | 91 |
|  |  | TRG |  |  |  |  |
|  | EXP.3 | 20℃/control | 17.478±0.446 | 0.981 | # | 67 |
|  | EXP.3 | 20℃/50μM | 17.188±0.545 |  |  | 64 |
|  |  | TRG |  |  |  |  |
|  | EXP.4 | 20℃/control | 17.719±0.416 | 0.191 | # | 96 |
|  | EXP.4 | 20℃/50μM | 18.231±0.492 |  |  | 91 |
|  |  | TRG |  |  |  |  |
|  | **CF1903 *glp-1(e2141)III.*** |  |  |  |  |  |
|  | EXP.1 | OP50(dead) |  |  |  |  |
|  | EXP.1 | 20℃/control | 30.789±1.077 | 0.539 | # | 90 |
|  |  | 20℃/50μM | 29.832±1.065 |  |  | 95 |
|  |  | TRG |  |  |  |  |
|  | EXP.2 | 20℃/control | 29.363±0.978 | 0.261 | # | 102 |
|  | EXP.2 | 20℃/50μM | 28.730±0.983 |  |  | 74 |
|  |  | TRG |  |  |  |  |
|  | EXP.3 | 20℃/control | 30.210±0.882 | 0.566 | # | 119 |
|  | EXP.3 | 20℃/50μM | 30.682±0.958 |  |  | 110 |
|  |  | TRG |  |  |  |  |
| 6F | **RSKS-1** |  |  |  |  |  |
|  | EXP.1 | OP50(dead) | 20.747±0.472 | 0.703 | # | 75 |
|  | EXP.1 | 20℃/control | 20.746±0.467 |  |  | 67 |
|  |  | 20℃/50μM |  |  |  |  |
|  |  | TRG |  |  |  |  |
|  | EXP.2 | 20℃/control | 20.455±0.483 | 0.778 | # | 66 |
|  | EXP.2 | 20℃/50μM | 20.302±0.504 |  |  | 63 |
|  |  | TRG |  |  |  |  |
|  | EXP.3 | 20℃/control | 20.500±0.594 | 0.565 | # | 82 |
|  | EXP.3 | 20℃/50μM | 20.235±0.570 |  |  | 81 |
|  |  | TRG |  |  |  |  |

*p*-value was analyzed by logRank Test.

N: number of dead worms.

*P* <0.05 indicated that the experiment was statistically significant, while *P* >0.05 indicated that the experiment was not statistically significant.

**Table S5. Effect of TRG on DAF-16** [**nuclear**](javascript:;) [**localization**](javascript:;)**and expression of HSP-4, HSP-6, HSP-60, GST-4 and SOD-3 in mutant nematode*.***

| Figure | Strain | Treatment | Mean±SEM | P value | N |
| --- | --- | --- | --- | --- | --- |
|  |  |  |  | VS |  |
|  |  |  |  | control |  |
|  |  |  |  |  |  |
| 3B | **SJ4100** | OP50(dead) |  |  |  |
|  | ***(zcIs13[hsp-6::GFP])*** |  |  |  |  |
|  | EXP.1 | 20℃/control | 4.792±0.120 | <0.0001 | 37 |
|  | EXP.1 | 20℃/50μM | 7.182±0.451 |  | 36 |
|  |  | TRG |  |  |  |
|  | EXP.2 | 20℃/control | 3.654±0.334 | <0.0001 | 33 |
|  | EXP.2 | 20℃/50μM | 7.240±0.649 |  | 31 |
|  |  | TRG |  |  |  |
|  | EXP.3 | 20℃/control | 3.311±0.217 | <0.0001 | 38 |
|  | EXP.3 | 20℃/50μM | 6.015±0.385 |  | 53 |
|  |  | TRG |  |  |  |
| 3D | **SJ4058** | OP50(dead) |  |  |  |
|  | ***(zcIs13[hsp-60::GFP])*** |  |  |  |  |
|  | EXP.1 | 20℃/control | 5.674±0.287 | 0.348 | 30 |
|  | EXP.1 | 20℃/50μM | 6.141±0.341 |  | 50 |
|  |  | TRG |  |  |  |
|  | EXP.2 | 20℃/control | 4.978±0.237 | 0.135 | 34 |
|  | EXP.2 | 20℃/50μM | 5.546±0.269 |  | 48 |
|  |  | TRG |  |  |  |
|  | EXP.3 | 20℃/control | 4.662±0.160 | 0.106 | 37 |
|  | EXP.3 | 20℃/50μM | 5.179±0.272 |  | 37 |
|  |  | TRG |  |  |  |
| 2B | **CF1553** | OP50(dead) |  |  |  |
|  | ***[(pAD76) sod-3::GFP*** |  |  |  |  |
|  | EXP.1 | 20℃/control | 3.279±0.150 | <0.0001 | 46 |
|  | EXP.1 | 20℃/50μM | 4.908±0.306 |  | 63 |
|  |  | TRG |  |  |  |
|  | EXP.2 | 20℃/control | 3.124±0.131 | <0.0001 | 55 |
|  | EXP.2 | 20℃/50μM | 4.793±0.182 |  | 34 |
|  |  | TRG |  |  |  |
|  | EXP.3 | 20℃/control | 3.647±0.204 | <0.0001 | 41 |
|  | EXP.3 | 20℃/50μM | 5.315±0.347 |  | 36 |
|  |  | TRG |  |  |  |
| 2C | **CL2166** |  |  |  |  |
|  | ***dvIs19(gst-4::GFP)*** | OP50(dead) |  |  |  |
|  | EXP.1 | 20℃/control | 5.172±0.216 | <0.0001 | 32 |
|  | EXP.1 | 20℃/50μM | 9.694±0.514 |  | 44 |
|  |  | TRG |  |  |  |
|  | EXP.2 | 20℃/control | 5.385±0.197 | <0.0001 | 38 |
|  | EXP.2 | 20℃/50μM | 9.060±0.385 |  | 37 |
|  |  | TRG |  |  |  |
|  | EXP.3 | 20℃/control | 4.938±0.242 | <0.0001 | 34 |
|  | EXP.3 | 20℃/50μM | 8.116±0.488 |  | 34 |
|  |  | TRG |  |  |  |
| 3C | **SJ4005** |  |  |  |  |
|  | ***zcIs4V(hsp-4::gfp)*** | OP50(dead) |  |  |  |
|  | EXP.1 | 20℃/control | 3.661±0.136 | 0.0001 | 39 |
|  | EXP.1 | 20℃/50μM | 4.598±0.182 |  | 48 |
|  |  | TRG |  |  |  |
|  | EXP.2 | 20℃/control | 3.978±0.189 | 0.1119 | 32 |
|  | EXP.2 | 20℃/50μM | 4.415±0.189 |  | 41 |
|  |  | TRG |  |  |  |
|  | EXP.3 | 20℃/control | 3.252±0.010 | <0.0001 | 45 |
|  | EXP.3 | 20℃/50μM | 4.845±0.155 |  | 54 |
|  |  | TRG |  |  |  |

*P* value was determined by comparison between the control group and the experimental group in each independent experiment. The data in the experimental group were counted as the number of normal dead nematodes. The experimental data were processed and analyzed by SPSS26.0 with Kaplan-Meier survival curve. P value was obtained by log-rank test analysis.

N: number of dead nematodes.

**Table S6. Effect of TRG on resistance to bacteria, high temperature and oxidation in wild-type nematode (N2).**

| Figure | Strain | Treatment | Mean lifespan  ±SEM  (Days) | P value  VS  control | change in mean lifespan | N |
| --- | --- | --- | --- | --- | --- | --- |
| 2A | **N2**  EXP.1  EXP.1 | **Paraquat(dead)**  20℃/control  20℃/50μM TRG | Day  4.452±0.190  5.770±0.207 | <0.0001 | 29.60% | 104  126 |
|  | EXP.2  EXP.2 | 20℃/control  20℃/50μM TRG | 4.714±0.176  5.705±0.208 | <0.0001 | 21.02% | 133  129 |
|  | EXP.3  EXP.3 | 20℃/control  20℃/50μM TRG | 4.111±0.228  5.333±0.299 | <0.0001 | 29.72% | 90  75 |
|  | **N2**  EXP.1  EXP.1 | OP50(dead)  35℃/control  35℃/50μM TRG | Hours  10.194±0.468  12.324±0.397 | 0.004 | 20.89% | 72  68 |
|  | EXP.2  EXP.2 | 35℃/control  35℃/50μM TRG | 10.200±0.429  11.180±0.484 | 0.102 | 9.60% | 70  61 |
|  | EXP.3  EXP.3 | 35℃/control  35℃/50μM TRG | 10.806±0.415  12.559±0.393 | 0.006 | 16.22% | 67  68 |
| 3E | **N2**  EXP.1  EXP.1 | **PA14**  20℃/control  20℃/50μM TRG | 16.384±0.537  19.478±0.564 | <0.0001 | 18.88% | 65  69 |
|  | EXP.2  EXP.2  EXP.3  EXP.3 | 20℃/control  20℃/50μM TRG  20℃/control  20℃/50μM TRG | 16.714±0.544  21.104±0.571  15.425±0.431  20.377±0.610 | <0.0001  <0.0001 | 26.26%  32.10% | 77  67  80  77 |

| Figure | Strain | Treatment | Mean±SEM | P value  VS  Control | N |
| --- | --- | --- | --- | --- | --- |
| 2D | **N2** | OP50(dead) |  |  |  |
|  | EXP.1  EXP.1  EXP.1  EXP.1 | 20℃/Control  20℃/50μM TRG  20℃/20mM Paraquat 20℃/ NAC | 8.117±0.740  3.400±0.181  13.306±0.809  3.289±0.136 | <0.0001  <0.0001  <0.0001 | 46  43  38  35 |
|  | EXP.2  EXP.2  EXP.2  EXP.2 | 20℃/Control  20℃/50μM TRG  20℃/20mM Paraquat 20℃/ NAC | 6.937±0.281  3.517±0.167  11.160±0.793  4.118±0.141 | <0.0001  <0.0001  <0.0001 | 37  41  32  36 |
|  | EXP.3  EXP.3  EXP.3  EXP.3 | 20℃/Control  20℃/50μM TRG  20℃/20mM Paraquat 20℃/ NAC | 7.433±0.464  3.853±0.112  12.100±0.899  4.435±.082 | <0.0001  <0.0001  <0.0001 | 41  37  40  44 |

On the 10th day, trigonelline-treated and untreated N2 nematodes were transferred into paraquat, 35℃ high temperature or *Pseudomonas aeruginosa* new plates, and the survival rate was calculated.

The experimental data were processed and analyzed by SPSS26.0, represented by Kaplan-Meier survival curve. *P* values were analyzed by Log-rank test.

**Table S7. Effects of TRG on age-related diseases.**

| Figure | Strain | Treatment | Mean±SEM  Days | P value  VS  6-OH DA | N |
| --- | --- | --- | --- | --- | --- |
| 4B | **BZ555**  ***egIs1(dat-1::gfp)*** | OP50(dead) |  |  |  |
|  | EXP.1  EXP.1  EXP.1  EXP.1 | 20℃/6-OH DA  20℃/6-OH DA+50μM TRG  20℃/Control  20℃/6-OH DA+L-DA | 7.302±0.346  19.795±1.354  22.971±1.267  20.515±1.307 | <0.0001  <0.0001  <0.0001 | 42  38  37  43 |
|  | EXP.2  EXP.2  EXP.2  EXP.2 | 20℃/6-OH DA  20℃/6-OH DA+50μM TRG  20℃/Control  20℃/6-OH DA+L-DA | 8.498±0.519  18.513±1.024  19.527±1.071  17.516±1.061 | <0.0001  <0.0001  <0.0001 | 32  47  39  38 |
|  | EXP.3  EXP.3  EXP.3  EXP.3 | 20℃/6-OH DA  20℃/6-OH DA+50μM TRG  20℃/Control  20℃/6-OH DA+L-DA | 5.335±0.323  11.814±0.616  11.322±0.437  12.897±0.812 | <0.0001  <0.0001  <0.0001 | 38  37  31  2 |

| 4A | **N5901 *Punc-54::α-Syn::YFP*** | OP50(dead) |  |  |  |
| --- | --- | --- | --- | --- | --- |
|  | EXP.1  EXP.1 | 20℃/control  20℃/50μM  TRG | 16.776±1.029  9.138±0.523 | <0.0001 | 33  31 |
|  | EXP.2  EXP.2 | 20℃/control  20℃/50μM  TRG | 14.954±0.670  8.138±0.555 | <0.0001 | 48  41 |
|  | EXP.3  EXP.3 | 20℃/control  20℃/50μM  TRG | 12.762±0.516  11.391±0.332 | <0.0001 | 43  52 |

AD

| Strain | Treatment | Mean lifespan  ±SEM | P value  VS  control | change in mean lifespan | N |
| --- | --- | --- | --- | --- | --- |
| CL4176  EXP.1  EXP.1 | OP50(dead)  20℃/control  20℃/50μM TRG | Hours  30.571±0.430  33.231±0.490 | <0.001 | 8.70% | 105  104 |
| EXP.2  EXP.2 | 20℃/control  20℃/50μM TRG | 30.082±0.462  32.333±0.454 | <0.05 | 7.48% | 98  96 |
| EXP.3  EXP.3 | 20℃/control  20℃/50μM TRG | 30.733±0.411  33.301±0.488 | <0.001 | 8.36% | 90  83 |
| CL2006  EXP.1  EXP.1 | OP50(dead)  20℃/control  20℃/50μM TRG | Days  16.444±0.434  19.066±0.535 | <0.001 | 15.95% | 63  61 |
| EXP.2  EXP.2 | 20℃/control  20℃/50μM TRG | 16.731±0.397  19.061±0.500 | <0.001 | 13.93% | 93  98 |
| EXP.3  EXP.3 | 20℃/control  20℃/50μM TRG | 15.909±0.636  19.115±0.762 | <0.001 | 20.15% | 66  61 |

HD

| Figure  4F | Strain | Treatment | Mean  Pigment±SEM | P value  VS  control | N | | Mean  Fluorescence  Intensity±SEM | P value  VS  control | N |
| --- | --- | --- | --- | --- | --- | --- | --- | --- | --- |
|  | N2 | OP50(dead) | 2 day of adult | | | | 4 day of adult | | |
|  | EXP.1  EXP.1 | 20℃/control  20℃/50μM  TRG | 19.303±1.105  17.639±1.235 | <0.050 | | 33  36 | 43.688± 1.273  36.700± 0.783 | <0.001 | 32  40 |
|  | EXP.2  EXP.2 | 20℃/control  20℃/50μM  TRG | 20.237±1.485  16.692±1.001 | <0.001 | | 38  39 | 39.576±1.426  32.500±0.785 | <0.001 | 33  38 |
|  | EXP.3  EXP.3 | 20℃/control  20℃/50μM TRG | 21.742±1.472  15.892±0.787 | <0.001 | | 31  37 | 41.484±1.181  33.829±0.751 | <0.001 | 31  35 |

| **Table S8. Effect of TRG on gene expression at mRNA level in nematode.** | | | | | |
| --- | --- | --- | --- | --- | --- |
|  |  |  |  |  |  |
| **Gene** | **EXP.1** | **EXP.2** | **EXP.3** | **Mean** | **SEM** |
|  |  |  |  |  |  |
| Control | 1 | 1 | 1 | 1 | 0 |
| *F08G5.6* | 1.437 | 1.652 | 1.25 | 1.447 | 0.095 |
| *F35E12.5* | 2.281 | 2.484 | 2.003 | 2.256 | 0.114 |
| *F55G11.4* | 1.351 | 1.284 | 1.22 | 1.285 | 0.031 |
| *irg-1* | 4.948 | 4.047 | 4.475 | 4.49 | 0.212 |
| *fard-1* | 1.274 | 2.108 | 1.846 | 1.743 | 0.201 |
| *daf-2* | 1.167 | 1.093 | 1.025 | 1.095 | 0.033 |
| *daf-16* | 0.72 | 0.858 | 0.751 | 0.776 | 0.034 |
| *sod-2* | 2.456 | 2.191 | 1.955 | 2.201 | 0.118 |
| *sod-3* | 1.418 | 1.473 | 1.079 | 1.323 | 0.101 |
| *dod-3* | 2.622 | 2.669 | 2.575 | 2.622 | 0.022 |
| *daf-9* | 2.621 | 1.915 | 2.24 | 2.259 | 0.166 |
| *hsp-12.6* | 2.152 | 2.164 | 2.177 | 2.164 | 0.006 |
| *hsp-16.1* | 1.767 | 2.746 | 2.34 | 2.284 | 0.232 |
| *hsp-60* | 1.092 | 1.081 | 1.07 | 1.081 | 0.005 |
| *skn-1* | 0.604 | 0.864 | 0.857 | 0.775 | 0.07 |
| *ctl-1* | 1.033 | 1.004 | 0.907 | 0.981 | 0.031 |
| *hsp-6* | 0.725 | 0.735 | 0.715 | 0.725 | 0.005 |
| *ctl-2* | 0.793 | 0.816 | 0.839 | 0.816 | 0.011 |
| *hsp-16.2* | 0.646 | 0.716 | 0.793 | 0.718 | 0.035 |
| *ctl-3* | 0.627 | 0.467 | 0.654 | 0.583 | 0.048 |

| gene | Forward primer | Reverse primer |
| --- | --- | --- |
| *cdc-42* | CTGCTGGACAGGAAGATTACG | CTCGGACATTCTCGAATGAAG |
| *F35E12.5* | ACACAATCATTTGCGATGGA | GGTAGTCATTGGAGCCGAAA |
| *T24B8.5* | AAACCTGTGGTGTCTGCGTTAC | TGGCAGGTTTTTGGGCATTG |
| *F08G5.6* | ATCGTTCCGAATGGTGGTTGAC | GCCGATTTCAGCTTGCAAAGTG |
| *F55G11.4* | GGATCCGTGTATTTGGCTGGAATCG | GTGAAGACATATGTGCTCCCGCGTT |
| *Y22F5A.5* | TGCTGATTTCCGTGCTTTCG | TTCCAACAGCATACACGTCACG |
| *irg-1* | AAGCAGCATGCGTATTTTCA | GCAGCTTCTCCTTTTTCTCC |
| *hsp-16.2* | CTGCAGAATCTCTCCATCTGAGTC | AGATTCGAAGCAACTGCACC |
| *hsp-16.1* | GTCACTTTACCACTATTTCCGTCCAGCTCAACGTTC | CAACGGGCGCTTGCTGAATTGGAATAGATCTTCC |
| *hsp-60* | AGGAGAAGCTTAATGAGCG | ACACGGTCCTTCTTCTCT |
| *hsp-12.6* | GTGATGGCTGACGAAGGAAC | GGGAGGAAGTTATGGGCTTC |
| *dve-1* | TCGAGGCCTCATACAAGAA | AAGAGGTTTTCCACAGTGTC |
| *hsp-6* | AGGAACAACAGAGTAAGATTTTC | TCGATTTGGTCCTTGGAAAG |
| *sod-3* | AGCATCATGCCACCTACGTGA | CACCACCATTGAATTTCAGCG |
| *nhr-57* | GACTCTGTGTGGAGTGATGGAGAG | GTGGCTCTTGGTGTCAATTTCGGG |
| *fard-1* | GGGTTTTTGGGAAAGGTGAT | CCACCGATTGCTTTCAATTT |
| *skn-1* | AGTGTCGGCGTTCCAGATTTC | GTCGACGAATCTTGCGAATCA |
| *daf-12* | AGGCGTTTCGTCAAAGTTGC | CCTGCTCTCCGAACAACGAT |
| *daf-9* | GAGGGCATTCTCCGCAAGT | CCACTGCTGAAGTCGAAATCC |
| *dod-3* | AAGCCATGTTCCCGAATGAG | GCTGCGAAAAGCAAGAAAATG |
| *F22B5.4* | GAGATCCACGTTTTGTTAAAGTCGC | CGGCGGACAAGGAATTGATAAGGAG |
| *daf-16* | CCAGACGGAAGGCTTAAAACT | ATTCGCATGAAACGAGAATG |
| *ctl-1* | GAATGTGAAGAATTATTTCGCTGA | AACTCGATTCCTGGGACGAT |
| *ctl-2* | CAAGGAACTACTTCGCTGAGG | AATGAGTGTCGGTGTACGAGAA |
| *ctl-3* | AGTAAATCTTCAAAATGCCAATG | GGTGGGGTTCCTGATTTCTAT |
| *sod-2* | GATACTGTCCAAAGGGAAAGAT | GTAGTAAGCGTGCTCCCAGA |
| *daf-2* | CGGTGCGAAGAGAGGATATT | TACAGAGGTCGCCGTTACTG |
| *gst-4* | TCCGTCAATTCACTTCTTCCG | AAGAAATCATCACGGGCTGG |
| *hsf-1* | TTGACGACGACAAGCTTCCAGT | AAAGCTTGCACCAGAATCATCCC |

**Table S9. Primers used for the analysis of mRNA expression levels in nematode.**
